# Identification of a new effector-immunity pair of *Aeromonas hydrophila* type VI secretion system

**DOI:** 10.1186/s13567-020-00794-w

**Published:** 2020-05-24

**Authors:** Shuiyan Ma, Yuhao Dong, Nannan Wang, Jin Liu, Chengping Lu, Yongjie Liu

**Affiliations:** 1 Joint International Research Laboratory of Animal Health and Food Safety, College of Veterinary Medicine, Nanjing Agricultural University, Nanjing, 210095 China; 2 College of Animal Science and Technology, Jinling Institute of Technology, Nanjing, 211169 China

## Abstract

The type VI secretion system (T6SS) is a multiprotein weapon that kills eukaryotic predators or prokaryotic competitors by delivering toxic effectors. Despite the importance of T6SS in bacterial environmental adaptation, it is still challenging to systematically identify T6SS effectors because of their high diversity and lack of conserved domains. In this report, we discovered a putative effector gene, U876-17730, in the whole genome of *Aeromonas hydrophila* NJ-35 based on the reported conservative domain DUF4123 (domain of unknown function), with two cognate immunity proteins encoded downstream. Phylogenetic tree analysis of amino acids indicates that AH17730 belongs to the Tle1 (type VI lipase effector) family, and therefore was named Tle1^AH^. The deletion of *tle1*^*AH*^ resulted in significantly decreased biofilm formation, antibacterial competition ability and virulence in zebrafish (*Danio rerio*) when compared to the wild-type strain. Only when the two immunity proteins coexist can bacteria protect themselves from the toxicity of Tle1^AH^. Further study shows that Tle1^AH^ is a kind of phospholipase that possesses a conserved lipase motif, Gly-X-Ser-X-Gly (X is for any amino acid). Tle1^AH^ is secreted by T6SS, and this secretion requires its interaction with an associated VgrG (valine-glycine repeat protein G). In conclusion, we identified a T6SS effector-immunity pair and verified its function, which lays the foundation for future research on the role of T6SS in the pathogenic mechanism of *A. hydrophila.*

## Introduction

*Aeromonas hydrophila* is a prevalent agent of aquatic infections, mainly causing motile aeromonad septicemia (MAS). As a food-borne pathogen, this organism represents a public health concern and causes soft-tissue wound infection and diarrhea [1]. Many virulence factors of *A. hydrophila* have been investigated, including motility [2], toxins [3], tissue-destructive enzymes [4] and S-layer [5]. Protein secretion systems are also essential for virulence and competition with nearby microorganisms. To date, nine types of bacterial secretion systems, from type I to type IX secretion systems (T1SS to T9SS), have been reported [6–8]. Among them, the type VI secretion system (T6SS) is one of the most commonly described secretion systems in Gram-negative bacteria.

T6SS was discovered as early as 2006 in *Pseudomonas aeruginosa* [9] and *Vibrio cholerae* [10], and approximately 25% of all Gram-negative bacteria have highly conserved T6SS gene clusters based on bioinformatics analysis [11]. T6SS is the main machinery that delivers antagonistic effector molecules into the environment, eukaryotic hosts and prokaryotic competitors for pathogenesis in a contact-dependent manner [12, 13]. The T6SS apparatus is composed of thirteen highly conserved “core” proteins and is believed to resemble the structure of the contractile tail of *Escherichia coli* bacteriophage T4 [14, 15]. Among the core components, TssB and TssC make up the bacteriophage contractile sheathe, which promotes the injection of a puncturing tube structure that comprises Hcp (hemolysin co-regulated protein) rings topped by a spike-like trimer of VgrG (valine-glycine repeat protein G). A previous study from Shneider et al. [16] showed that PAAR (proline-alanine-alanine-arginine) repeat-containing proteins bind to the ends of endogenous VgrG proteins. Another important component, ClpV, represents the central energy source for T6SS and mediates remodeling of the VipA/VipB cogwheel-like protein complex, which is conserved and essential for T6SS function [17]. Among these “core” proteins, Hcp and VgrG are the most studied components. Additional copies of these two proteins can also be found outside of the main T6SS cluster, generally around genes encoding putative effector proteins, and are used as chaperones for the secretion of effectors [18, 19]. Effector proteins are delivered through the T6SS and injected into target cells in a one-step manner. The known T6SS effectors could be transported either fused to structural components (specialized effectors) or via direct interactions with one protein of the core complex (cargo effectors) [20]. Meanwhile, there are corresponding antagonistic immunity genes located downstream of each effector gene to neutralize toxic effectors, preventing self-killing or sibling-intoxication, and thus a new toxin-antitoxin module called the effector-immunity (E-I) pair was constituted [21–23]. Although diverse approaches have been used to identify T6SS effectors, the number of identified effectors remains limited. The most common method to identify effectors is comparing the secretomes of WT (wild type) and T6SS mutants by transcriptomics, mutant library screening or proteomics-based methods [24]. In recent years, bioinformatics analysis has been used more frequently to predict effector genes by identifying highly conservative domains, such as the common N-terminal MIX motif, and DUF4123 and DUF2169 proteins [25–27]. However, due to the diversity of bacteria and the complexity of effector proteins, systematic identification of T6SS effectors is still a challenge.

In this study, we report the identification of a novel T6SS effector, Tle1^AH^ (type VI lipase effectors of *A. hydrophila*), based on the conserved domain DUF4123, that plays a critical role in the successful colonization of *A. hydrophila* in the host. Two immune proteins work together to protect bacteria from the toxicity of Tle1^AH^. Further study found that the secretion of Tle1^AH^ is associated with an interaction with VgrG but not Hcp. The present findings will provide valuable insights into the role of T6SS in *A. hydrophila*.

## Materials and methods

### Ethics statement

Zebrafish (*Danio rerio*) and crucian carp (*Carassius auratus*) were obtained from the Pearl River Fishery Research Institute, Chinese Academy of Fishery Science. The animal experiments were performed according to the Ethical Committee for Animal Experiments of Nanjing Agricultural University, China [permit number: SYXK (SU).2017-0007]. All operations are in line with the guidelines of the Animal Welfare Council of China.

### Bacterial strains, plasmids and growth conditions

The bacterial strains and plasmids used in this study are listed in Table 1. Strains were grown at 28 °C or 37 °C in Luria–Bertani (LB; 10 g/L tryptone, 5 g/L yeast extract, 10 g/L NaCl) media. When required, the final concentrations of antibiotics in growth media were as follows: 34 µg/mL chloramphenicol (Cm, in absolute ethanol), 100 μg/mL ampicillin (Amp, in water), 50 μg/mL kanamycin (Kan, in water) and 20 µg/mL gentamicin (Gm, in water). All antibiotics were stored at 4 °C.

**Table 1:** Strains used in this study

Strain or plasmid	Description	Source
*Strain*		
*A. hydrophila* NJ-35	Wilde-type, isolated from diseased crucian carp, in China, Amp^r^	Laboratory stock
*A. hydrophila* J-1	Wilde-type, isolated from diseased crucian carp, in China, Amp^r^	Laboratory stock
*A. hydrophila* NJ-3	Wilde-type, isolated from pond water, in China	Laboratory stock
*A. hydrophila*ATCC 7966	Wilde-type, isolated from fishy milk, in USA	Laboratory stock
*A. salmonicida* CS-2	Wilde-type, isolated from pond water, in China	Laboratory stock
*A. media* NJ-8	Wilde-type, isolated from pond water, in China	Laboratory stock
*A. veronii* XH-14	Wilde-type, isolated from diseased Chinese bream, in China	Laboratory stock
*V. parahaemolyticus* RIMD 2210633	Wilde-type, isolated from a patient suffering from diarrhoea, in Japan	Laboratory stock
*E. coli* SM10	λpir^+^, Kan^r^	[28]
*E. coli* BL21	*E. coli* strain, competent invitrogen cells	CWBIO
*E. coli* TOP10	*E. coli* strain, competent invitrogen cells	CWBIO
∆*tle1*^*AH*^	*tle1*^*AH*^ gene deletion mutant from NJ-35	This study
Δ*clpV*	*clpV* gene deletion mutant from NJ-35	[32]
∆*tle1*-*tli1tli2*^*AH*^	*tle1*-*tli1tli2*^*AH*^ gene deletion mutant from NJ-35	This study
C∆*tle1*^*AH*^	∆*tle1*^*AH*^ with the vector pMMB-*tle1*^*AH*^	This study
C∆*tli1*^*AH*^	∆*tle1*-*tli1tli2*^*AH*^ with the vector pMMB-*tli1*^*AH*^	This study
C∆*tli2*^*AH*^	∆*tle1*-*tli1tli2*^*AH*^ with the vector pMMB-*tli2*^*AH*^	This study
C∆ *tli1tli2*^*AH*^	∆ *tle1*-*tli1tli2*^*AH*^ with the vector pMMB-*tli1tli2*^*AH*^	This study
*Plasmid*		
pYAK1	R6K-ori suicide vector, SacB^+^, Cm^r^	[27]
pYAK1-*tle1*^*AH*^	pYAK1 carrying the flanking sequence of *tle1*^*AH*^, Cm^r^	This study
pYAK1-*tle1*-*tli1tli2*^*AH*^	pYAK1 carrying the flanking sequence of *tle1*^−^*tli1tli2*^*AH*^, Cm^r^	This study
pMMB207	Low-copy-number vector, Cm^r^	[29]
pMMB-*tle1*^*AH*^	pMMB207 carrying the *tle1*^*AH*^ coding region, Cm^r^	This study
pMMB-*tli1*^*AH*^	pMMB207 carrying the *tli1*^*AH*^ coding region, Cm^r^	This study
pMMB-*tli2*^*AH*^	pMMB207 carrying the *tli2*^*AH*^ coding region, Cm^r^	This study
pMMB-*tli1tli2*^*AH*^	pMMB207 carrying the *tli1tli2*^*AH*^ coding region, Cm^r^	This study
pMMB- *tle1*^*AH*^(6*His)	pMMB207 carrying the *tle1*^*AH*^+ 6*His coding region, Cm^r^	This study
pMMB-Kan	pMMB207 carrying the Kan coding region, Cm^r^, Kan^r^	This study
pMMB-Gen	pMMB207 carrying the Gen coding region, Cm^r^, Ge^r^	This study
pBAD/HisA	Expression vector, Amp^r^	Invitrogen
pBAD-*tle1*^*AH*^	pBAD/HisA carrying *tle1*^*AH*^ sequence	This study
pBAD-peri-*tle1*^*AH*^	pBAD/HisA carrying peri + *tle1*^*AH*^ sequence, peri is the PelB leader sequence	This study
pBAD-peri-*tle1*^*AHS303A*^	pBAD/HisA carrying peri + *tle1*^*AHS303A*^ sequence, peri is the PelB leader sequence	This study
pGEX-4T-1	Expression vector, Amp^r^	Invitrogen
pGEX-*tle1*^*AH*^	pGEX-4T-1 carrying *tle1*^*AH*^ sequence	This study
pET-28a	Expression vector, Kan^r^	Invitrogen
His-*vgrG*	pET-28a carrying *vgrG* sequence	This study
His-*hcp*	pET-28a carrying *hcp* sequence	This study

All reagents used in this study were supplied by Sigma (St. Louis, MO, USA) unless otherwise indicated.

### Construction of gene deletion mutants and *A. hydrophila* complementation strains

Single or double gene mutants were constructed by homologous recombination using the suicide plasmid pYAK1 [28]. All oligonucleotide primers are listed in Additional file 1. The left and right flanking regions of the target gene were PCR-amplified and ligated in-frame using fusion PCR. Then, the fusion fragments were cloned into pYAK1 with the restriction enzyme BamHI and then chemically transformed into *E. coli* SM10 competent cells [29]. For conjugation, the recombinant *E. coli* SM10 (Cm resistant, Cm^r^) and *A. hydrophila* NJ-35 (Amp resistant, Amp^r^) grown to log phase were mixed at a ratio of 3:1 (vol/vol) and then spotted on a 0.22-µm nylon filter (OKLABS, Tianjin, China) on LB plates. After incubation at 28 °C for 20 h, the bacteria were washed from the filter and grown on LB plates containing Amp and Cm. The positive colonies were verified by PCR and cultured in LB medium without NaCl for three generations. Then, the double crossover mutants were selected on LB agar plates containing 20% sucrose. The final mutants were confirmed by PCR amplification for the deleted region and flanking DNA, followed by DNA sequencing. In the same way, a triple-deletion mutant was also constructed.

The corresponding complemented strains of the mutants were constructed with the shuttle plasmid pMMB207 [30]. The target genes were amplified from the chromosomal DNA of *A. hydrophila* NJ-35 and then ligated into the pMMB207 vector. The recombinant plasmids were transformed into *E. coli* SM10 to serve as a donor strain, and then transferred into the mutant strains by conjugation as above. The transconjugants were selected on LB agar plates containing Amp and Cm and further confirmed by PCR.

### Growth curve determination

*Aeromonas hydrophila* strains were cultured in LB broth at 28 °C until OD_600_ values were up to 0.5 (2.5 × 10^8^ CFU/mL, as determined by counting the number of colony forming units (CFU) on agar plates). Then, the bacteria in the early logarithmic stage of growth were inoculated into flasks (OKLABS) containing 25 mL of fresh LB medium at 1:100. The flasks were placed in a shaker at 28 °C and incubated for 16 h. Every 1 h, the OD_600_ was measured using a SmartSpec Plus spectrophotometer (BIO-RAD, USA). The experiment was repeated three times independently.

### Biofilm formation assay

Biofilm formation was assayed as previously described [31] with some modification. In brief, *A. hydrophila* strains were cultured in LB broth at 28 °C to an OD_600_ of 0.6–0.8 and normalized to an OD_600_ of 0.1. Then, 200 µL aliquots of bacterial suspensions (1:100 dilution in LB) were dispensed into 96-well polystyrene plates (Beyotime, Shanghai, China), and an equal volume of fresh medium was used as the blank control. Each strain was replicated in eight wells. To avoid edge effect resulting from evaporation and temperature fluctuation, no samples were added in the outermost two rows and two columns of the 96-well plates. After the plates were incubated at 28 °C for 24 h without shaking, the culture supernatant was discarded, and the wells were washed three times with sterile phosphate buffered saline (PBS) by removing the fluid with pipettors. After air-drying for 15 min, 200 µL methanol per well was added, and adherent bacterial cells were fixed for 15 min. Then, methanol was removed, and the wells were dried for 15 min. The attached bacteria were stained with 200 μL 1% crystal violet solution for 10 min. The plates were then washed with double deionized water (ddH_2_O) five times as above, followed by air-drying for 10 min. Subsequently, the bound crystal violet was solubilized using absolute ethanol, and the OD_595_ value of each well was measured. The assay was performed in three independent experiments.

### Antibacterial competition assay

Competition experiments were performed as previously described [32] with some modifications. To construct a T6SS^−^ strain, we inactivated the *clpV* gene, which encodes a putative ATPase required for T6SS function [33]. *A. hydrophila* NJ-35 and its derived strains were used as the predator strains, while *E. coli* BL21, *V. parahaemolyticus* RIMD 2210633, and other *Aeromonas* strains (Table 1) served as the preys. The predator and prey strains were cultured for 5 h, respectively; the cultures were adjusted to an OD_600_ of 1.0 and concentrated 10 times. Cells were mixed together at a ratio of 5:1 (predator to prey); 25 μL of the mixture was spotted onto a 0.22-μm nylon filter on LB plates, and the plates were incubated for 3 h at 28 °C. Prey cells that were mixed with an equal volume of LB media were used as a control. Then, the cultures on the spots were suspended in 1 mL of LB broth. The CFU of surviving prey cells were enumerated by serial dilution and plating onto the correspondingly selective medium. The experiment was repeated three times independently.

### Protein secretion assay

A protein secretion assay was performed to explore the secretion of Tle1^AH^ in *A. hydrophila* as described elsewhere [26]. The *tle1*^*AH*^ gene was expressed in *A. hydrophila* through the recombinant plasmid pMMB207-*tle1*^*AH*^ (fused with 6_*_His-tag). Then, the strains were grown in 500-mL glass culture flasks with 200 mL LB medium for 18 h, and bacterial cultures were collected by centrifugation at 10 000 × *g* for 10 min. The cell pellets were resuspended using 5 mL PBS and 50 µL 5 × SDS-PAGE (sodium dodecyl sulfate/polyacrylamide gel electrophoresis; KeyGEN BioTECH, Nanjing, China) sample loading buffer. The culture supernatants were filtered using a 0.22-µm membrane filter (OKLABS), and 100% ice-cold trichloroacetic acid (TCA) solution (to 10%) was added to precipitate proteins on ice for 1 h. The proteins were then centrifuged at 15 000 × *g* for 15 min at 4 °C, and the supernatant was discarded. The concentrated protein precipitate was washed twice with 100% acetone and centrifuged at 15 000 × *g* for 10 min. After air-drying in a sterile laminar flow hood for 20 min, the proteins were collected and treated with 5× SDS-PAGE buffer. Then, the protein samples from cell pellets and culture supernatants were analyzed by SDS-PAGE and Western blot with anti-His mouse monoclonal antibody (mAb; Abmart, Shanghai, China) or anti-GroEL (heat shock protein Hsp60) polyclonal antiserum [34]. Here GroEL (a cytoplasmic protein) serves as a loading control.

### Growth curves for Tle1^AH^ toxicity assays

The Tle1^AH^ toxicity assay was carried out as described previously [35]. pBAD/HisA (Invitrogen) was used for construction of the expression vectors for *tle1*^*AH*^ and its point mutant *tle1*^*AHS303A*^ (the catalysis site of Tle1^AH^ at position 303 mutated from serine to alanine). Point mutation S303A was generated by fusion PCR and further verified by sequencing [34]. To achieve periplasmic localization, the PelB leader sequence [36] was fused in front of the *tle1*^*AH*^ and *tle1*^*AHS303A*^*. E. coli* TOP10 containing pBAD/HisA–*tle1*^*AH*^ or pBAD/HisA–*tle1*^*AHS303A*^ were grown overnight at 37 °C in a 5-mL eppendorf tube (GeGene Tech, Shanghai, China) with LB medium containing Amp; the OD_600_ was adjusted to 0.5, and the cultures were inoculated into a 50-mL glass flask with 20 mL LB broth at a ratio of 1:100. Cultures were induced with 0.25% l-arabinose after 1.5 h of growth. A growth curve was drawn by measuring the OD_600_ every 30 min. The experiment was repeated three times independently.

### Expression and purification of proteins

Primers were designed according to the sequences of the *tle1*^*AH*^ and *vgrG* genes of *A. hydrophila* NJ-35 in GenBank (accession number NZ_CP006870). *Tle1*^*AH*^ was cloned into the pGEX-4T-1 vector (Invitrogen) for expression with an N-terminal glutathione-S-transferase (GST) tag. *VgrG/hcp* was cloned into the pET-28a vector (Invitrogen) with a His tag. The GST-Tle1^AH^ and His-VgrG/Hcp proteins were expressed in BL21 (DE3) cells (CWBIO, Beijing, China). The transformed cells were cultured in LB medium at 37 °C to an OD_600_ of 0.8, at which time the fusion protein expression was at the highest level and most was expressed as soluble protein not as an inclusion body based on our preliminary experiment. Protein expression was induced with 100 mM isopropyl β-d-1-thiogalactopyranoside (IPTG) at 16 °C for 20 h. The cultures were harvested by centrifugation at 8000 × *g*, resuspended in 1 × PBS and lysed by sonication. The lysate was centrifuged at 12 000 × *g* for 15 min at 4 °C to remove precipitate, and then the supernatant was loaded on a Ni^2+^-NTA column (GE Healthcare, Shanghai, China) to purify the proteins. The eluted proteins were collected and dialyzed for pull-down assays.

### GST pull-down assay

A GST pull-down assay was employed to identify the interactions between Tle^AH^ and VgrG/Hcp. Briefly, GST-Tle1^AH^ proteins were incubated with prepared glutathione Sepharose beads (25 µm; Enriching Biotechnology, Shanghai, China) on a rotating incubator for 3 h at 4 °C, and then the beads were collected and washed three times with PBS (pH 7.4). Then, His-VgrG/Hcp proteins were added to the beads and incubated for 3 h at 4 °C. The beads were washed with PBS buffer five times, and the bound proteins were washed off the beads with elution buffer (50 mM Tris–HCl, 10 mM GSH, pH 8.0). The elution was analyzed and detected by SDS-PAGE and Western blotting with anti-His or anti-GST mouse mAb (Abmart).

### Western blotting analysis

Protein samples with SDS loading buffer were boiled for 10 min; 10 μL of each sample was loaded on an SDS-PAGE gel, which was run at 80-120 V by Bio-Rad PowerPac Basic for 1 h and transferred to a 0.22-µm NC (nitrocellulose) filter membrane (Solarbio, Beijing, China) by electroblotting apparatus (Bio-Rad, USA). The membrane was blocked with 5% (wt/vol) skimmed milk in TBST (20 mM Tris–HCl, 150 mM NaCl, 0.05% (V/V) Tween 20) buffer for 2 h at 37 °C, incubated with anti-His, anti-GST or anti-GroEL antibody (1:5000) for 1.5 h at room temperature and washed three times with TBST buffer, incubated with horseradish peroxidase (HRP)-conjugated goat anti-mouse IgG or goat anti-rabbit IgG antibody (1:5000; Linc-Bio Science, Shanghai, China) for 1.5 h, and washed three times in TBST buffer. The blots were then detected using the Enhanced Chemiluminescence (ECL) Detection Kit (CMCTAG, USA) and ChemiDocTM Touch imaging system (Bio-Rad, USA).

### Determination of the bacterial median lethal dose (LD_50_)

A LD_50_ challenge assay was performed as previously reported [33]. Briefly, the mid-logarithmic bacterial cultures were washed three times with sterile PBS and serially diluted tenfold from 5 × 10^6^ to 5 × 10^1^ CFU/mL. For each *A. hydrophila* strain, six groups of 10 zebrafish per group were intraperitoneally (i.p.) injected with 20 µL of the bacterial suspension in PBS. Additionally, 10 zebrafish that were injected only with PBS served as a negative control. Mortality was recorded twice per day for 7 days, and the LD_50_ values were calculated following the method of Reed and Muench [37].

### Tissue colonization by wild-type and *tle1*^*AH*^ mutant strains

A competitive colonization assay was performed as previously reported [35]. To assay the competitive colonization of NJ-35 and its mutant derivatives in heart, hepatopancreas, spleen and kidney of crucian carp, WT and mutant bacteria were suspended in PBS to achieve a final concentration of 6.5 × 10^7^ CFU/mL, respectively and mixed at a 1:1 ratio. Then, 100 µL of the mixture was intraperitoneally injected into five fish. After 24 h, the organ samples were collected in a sterile environment, homogenized by vortexing in 900 µL of PBS, and diluted tenfold in PBS; aliquots were plated onto LB agar with the corresponding antibiotics. The bacteria used here carried either a Gm or Kan resistance vector, allowing them to be easily screened. For graphical and statistical purposes, the viable plate counts (CFU per gram of sample) were log_10_ transformed.

### Bioinformatics analysis

Nucleotide and protein sequences were acquired from the National Center for Biotechnology Information database [38]. Phylogenetic tree reconstruction was performed using MEGA 7.0 [39], and sequence logos were generated using Geneious Prime 2019.

### Statistical analyses

Data were analyzed and plotted using GraphPad Prism version 7 software. Multiple comparisons were performed by analysis of variance (ANOVA) followed by the Turkey multiple-comparison test. *P*-values < 0.05 were considered to be statistically significant. The error bars presented in the figures represent the standard deviations of the means of multiple replicate experiments.

## Results

### A potential T6SS effector is predicted in *A. hydrophila* NJ-35

**Figure 1:**
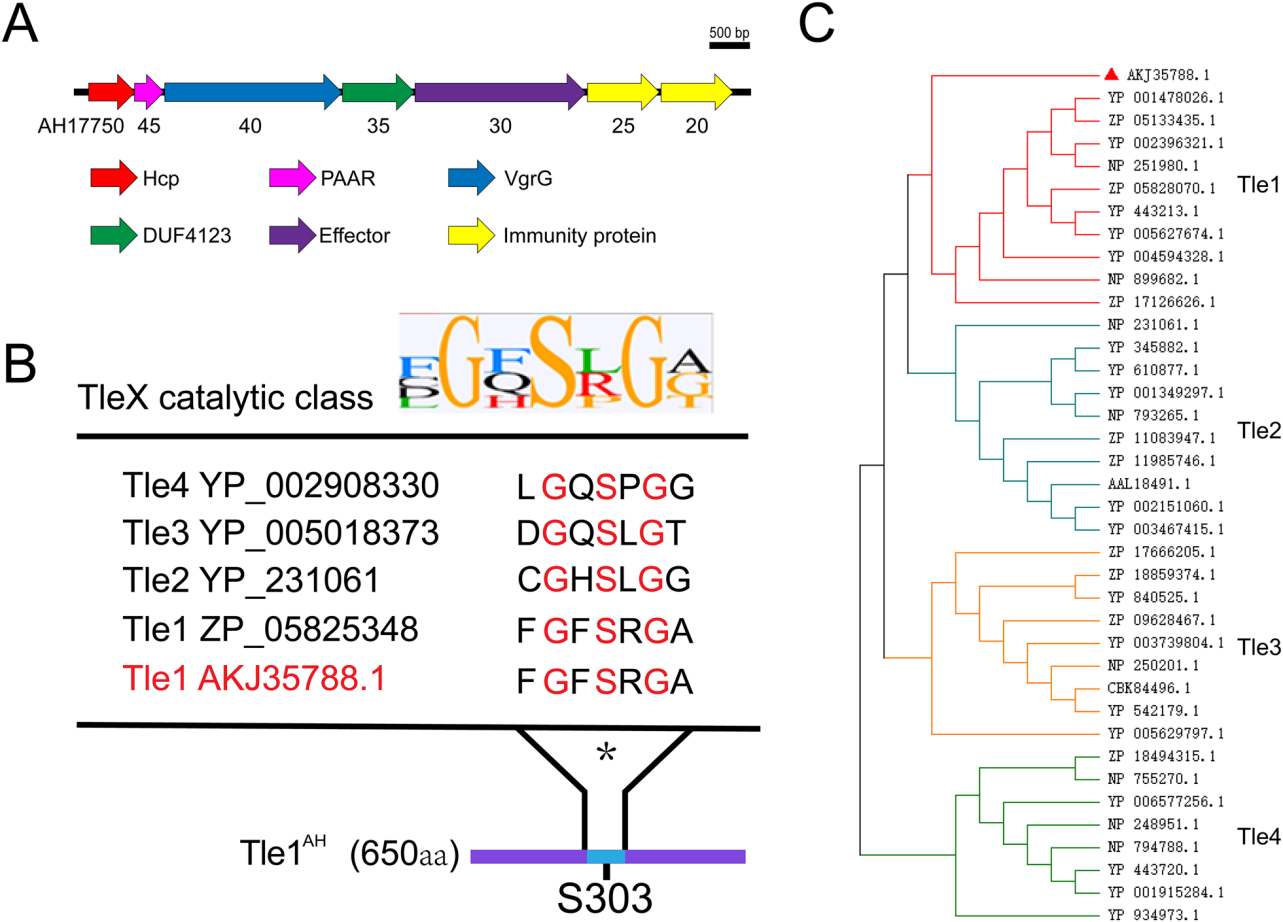
**Tle1**^**AH**^**is a potential T6SS effector in*A. hydrophila*NJ-35. A** Genetic organization of T6SS-related proteins containing the DUF4123 domain in *A. hydrophila* NJ-35. The numbers below refer to the gene locus tag (U876-XXXXX). Sequencing data for NJ-35 can be obtained from the National Center for Biotechnology Information (accession number: CP006870). **B** Sequence alignment of conserved catalytic motifs (labeled in red) compared between Tle families. Sequence logos were generated from alignments of the catalytic motifs from the families Tle1-4 (Gly**-**X-Ser-X-Gly, X is for any amino acid). * represents the catalytic residues. **C** Phylogenetic analyses of Tle1^AH^ (AKJ35788.1) with representative members of the families Tle1-4. Figure was prepared using MEGA7.0.

Based on the conserved domain DUF4123 [26], we searched potential T6SS effectors in *A. hydrophila* NJ-35. A putative effector-immunity (AH17720-17730) pair encoded downstream of the DUF4123 (AH17735) domain was predicted (Figure 1A). Bioinformatics analysis indicated that the AH17730 protein possesses a highly conserved DUF2235 domain, with a catalytic motif Gly-X-Ser-X-Gly (X is for any amino acid), which is common in esterases and lipases (Figure 1B). Further phylogenetic analysis suggested that AH17730 belongs to the Tle1 family (Figure 1C); therefore, AH17730 was named Tle1^AH^.

### Tle1^AH^ mediates interbacterial antagonism

**Figure 2:**
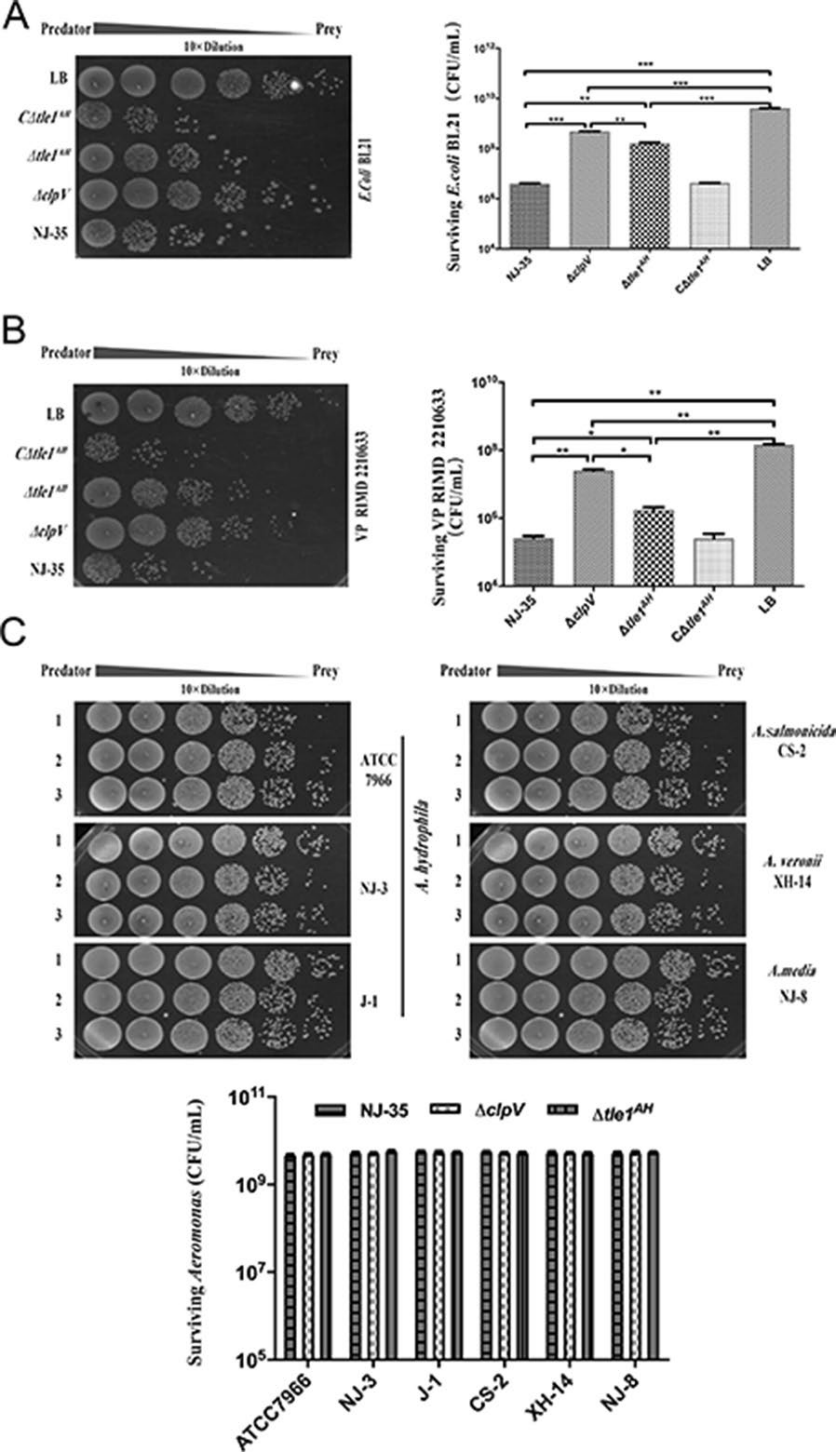
**Tle1**^**AH**^**is required for the interbacterial antagonism of*A. hydrophila*NJ-35.** Predator and prey cells at a ratio of 5:1 were cocultured to assay the recovery of surviving prey cells by determining colony forming unit (CFU). *A. hydrophila* NJ-35 and its mutant derivatives ∆*clpV*, ∆*tle1*^*AH*^ or C∆*tle1*^*AH*^ were used as the predator strains. *ClpV*, which encodes a putative ATPase required for T6SS function, was deleted to construct the T6SS^−^ strain (∆*clpV*). “LB” indicates incubation of *E. coli* with sterile LB medium alone and serves as the control. **A***E. coli* BL21 as the prey strain. **B***V. parahaemolyticus* RIMD 2210633 as the prey strain. **C***Aeromonas* strains as the preys, including *A. hydrophila* strains ATCC 7966, J-1 and NJ-3, *A. sobria* CS-2, *A. media* NJ-8 and *A. veronii* XH-14. Lane 1, the wild-type *A. hydrophila* NJ-35; Lane 2, ∆*clpV* (T6SS^−^); Lane 3, ∆*tle1*^*AH*^. Data are presented as the mean ± standard deviation (error bars) of three independent experiments. ***P < 0.001, **P < 0.01. *P < 0.05.

To determine whether *tle1*^*AH*^ participates in inter-generic antagonism of *A. hydrophila* NJ-35, we performed a quantitative bacterial competition assay using *E. coli* BL21 and *V. parahaemolyticus* RIMD 2210633 as prey strains. The CFU of surviving prey cells were enumerated on LB agar containing Kan. As shown in Figure 2A, compared with the LB group (without the predator), the wild-type NJ-35 and its derived mutants ∆*tle1*^*AH*^ and ∆*clpV* (T6SS^−^) caused a considerable reduction in survival of *E. coli* (*P *< 0.001). Mutant strains ∆*tle1*^*AH*^ (*P *< 0.01) and ∆*clpV* (*P *< 0.001) exhibited significantly decreased killing ability compared to the wild-type strain, while the capacity of the former to kill *E. coli* was stronger than the latter (*P *< 0.01). The killing ability of the complement strain C∆*tle1*^*AH*^ was restored to the wild-type level. Similar results to *E. coli* were obtained for *V. parahaemolyticus* (Figure 2B).

Further, we evaluated the intra-generic role of *tle1*^*AH*^ by examining the abilities of *A. hydrophila* NJ-35 and its derived deletion mutant ∆*tle1*^*AH*^ to outcompete other *A. hydrophila* strains (ATCC 7966, J-I and NJ-3) or related species including *A. salmonicida* CS-2, *A. media* NJ-8 and *A. veronii* XH-14. Notably, no significant difference was observed in CFU of surviving *Aeromonas* when the wild-type NJ-35, ∆*tle1*^*AH*^ or ∆*clpV* were used as the predator strains (Figure 2C). These data suggest that Tle1^AH^ may be involved in inter-generic but not intra-generic bacterial competition of *A. hydrophila* NJ-35.

### Tle1^AH^ is a phospholipase effector secreted by T6SS

**Figure 3:**
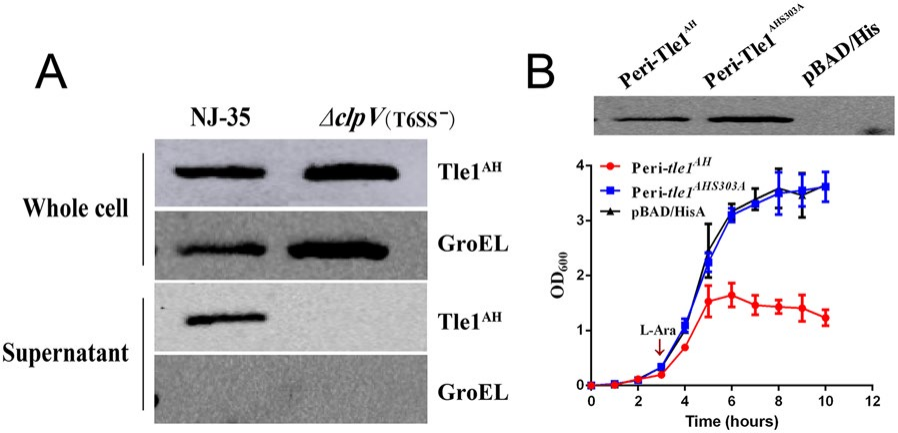
**Tle1**^**AH**^**is a phospholipase effector secreted by T6SS of*A. hydrophila*NJ-35**. **A** T6SS-dependent secretion of Tle1^AH^ was confirmed by Western blot on whole cells and supernatants of *A. hydrophila* NJ-35 and the ∆*clpV* strain. *ClpV*, which encodes a putative ATPase required for T6SS function, was deleted to construct the T6SS^−^ strain (∆*clpV*). The anti-His antibody was used to measure the production of Tle1^AH^ and anti-GroEL antibody served as an internal reference. GroEL: heat shock protein Hsp60. **B** Growth of *E. coli* TOP10 producing peri-Tle1^AH^ and peri-Tle1^AHS303A^ in LB broth. pBAD/His was used for construction of the expression vectors for *tle1*^*AH*^ and its point mutant *tle1*^*AHS303A*^ (the catalysis site of Tle1^AH^ at position 303 mutated from serine to alanine). To achieve periplasmic localization, the PelB leader sequence was fused in front of the *tle1*^*AH*^ and *tle1*^*AHS303A*^. Cultures were induced by l-arabinose (l-Ara) at the indicated time by the arrow. A growth curve was drawn by measuring the OD_600_ every 30 min. Data are presented as the mean ± standard deviation (error bars) of three independent experiments. The expression of peri-Tle1^AH^ and peri-Tle1^AHS303A^ was detected in *E. coli* TOP10 by Western blot using anti-His antibody.

To determine if the secretion of Tle1^AH^ is accomplished via T6SS in *A. hydrophila* NJ-35, we constructed a pMMB-*tle1*^*AH*^ overexpression vector labeled with a 6_*_His-tag. Then, the recombinant vector from the donor strain *E. coli* SM10 was conjugated into the recipient strains NJ-35 and ∆*clpV*. Western blot analysis showed that Tle1 protein could be detected in the supernatant of the wild-type strain NJ-35 but not in that of the ∆*clpV* mutant strain. For reference, Tle1^AH^ was detected in the whole cells of both strains (Figure 3A). The results demonstrate that the secretion of Tle1 protein depends on T6SS.

We reasoned that if Tle1^AH^ is a phospholipase with the Gly-X-Ser-X-Gly sequence between amino acids 303 and 307 as the catalytic motif and is secreted by T6SS, a point mutation of the catalytic motif will abolish its enzymatic activity. To prove its catalytic activity, we mutated the serine catalysis site (S303A) and constructed expression vectors for *tle1*^*AH*^ and its point mutant *tle1*^*AHS303A*^. Western blot analysis indicates that peri-*tle1*^*AH*^ and peri-*tle1*^*AHS303A*^ recombinant vectors were well expressed in *E. coli* TOP10 cells. The growth curve indicates an obvious decrease in the survival of *E. coli* containing the peri-*tle1*^*AH*^ recombinant plasmid, while the *E. coli* containing peri-*tle1*^*AHS303A*^ plasmid shows a similar growth rate as the negative control bacteria (Figure 3B). The results proved that Tle1^AH^ is a T6SS phospholipase effector and toxic to *E. coli* when artificially localized to the periplasm via a sec-dependent leader sequence.

### Tle1^AH^ and Tli1Tli2^AH^ are an effector-immunity pair

**Figure 4:**
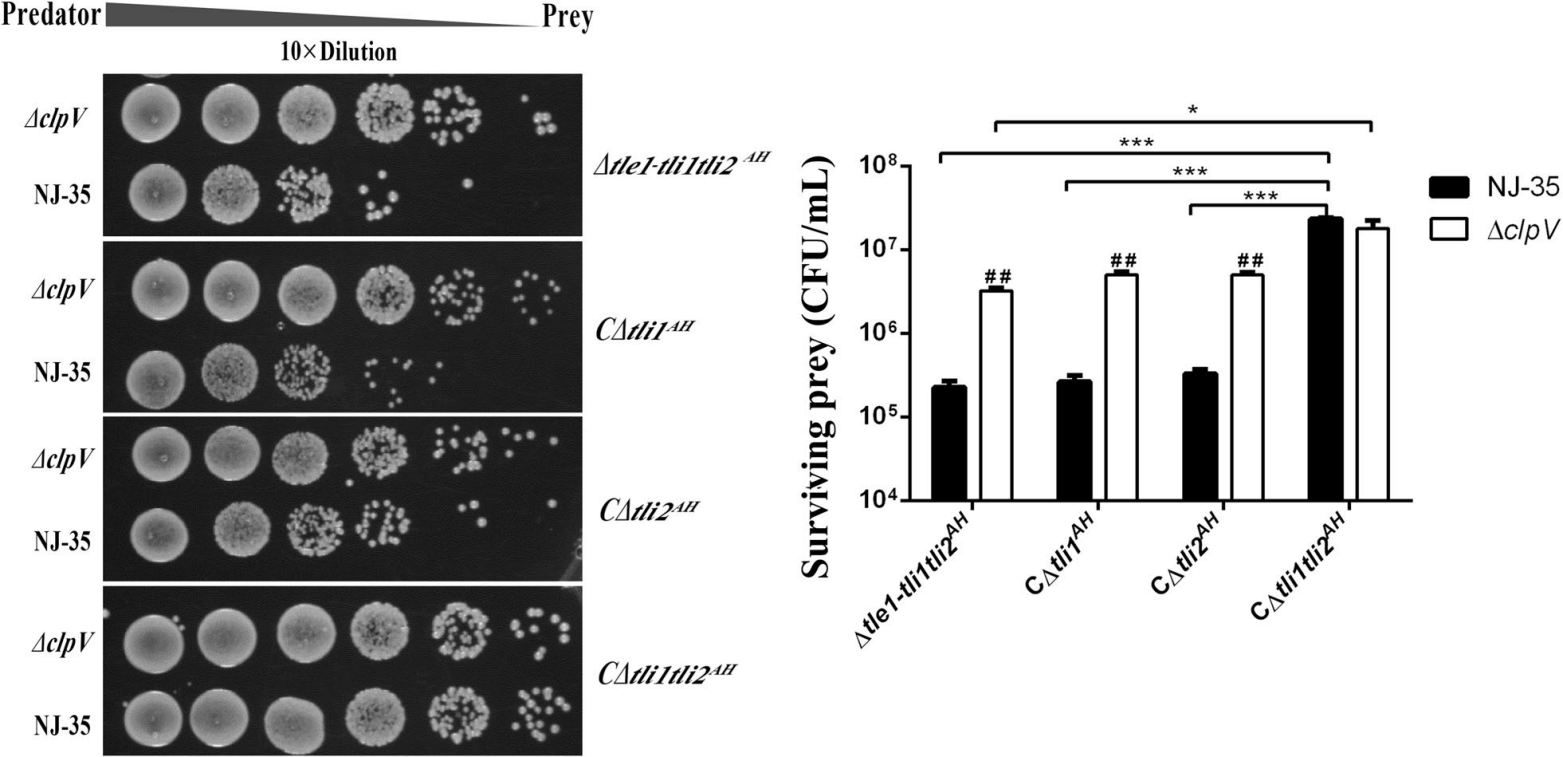
**Tli1Tli2**^**AH**^**are the cognate immunity proteins to Tle1**^**AH**^. *A. hydrophila* NJ-35 and the ∆*clpV* strain were used as the predator strains. *ClpV*, which encodes a putative ATPase required for T6SS function, was deleted to construct the T6SS^−^ strain (∆*clpV*). The prey strains included the gene deletion mutant ∆*tle1*-*tli1tli2*^*AH*^ and its single or double restoration strains of immunity genes, they are, C∆*tli1*^*AH*^ (∆*tle1*-*tli1tli2*^*AH*^*/*pMMB-*tli1*^*AH*^), C∆*tli2*^*AH*^ (∆*tle1*-*tli1tli2*^*AH*^*/*pMMB-*tli2*^*AH*^) and C∆*tli1tli2*^*AH*^ (∆*tle1*-*tli1tli2*^*AH*^*/*pMMB-*tli1tli2*^*AH*^). The predator and prey strains were cultured at a ratio of 5:1, and surviving prey cells were serially diluted and determined on the LB plate containing antibiotics. Data are presented as the mean ± standard deviation (error bars) of three independent experiments. ^##^*P* < 0.01 indicates a significant difference between this group and the NJ-35 group. ***P < 0.001 or *P < 0.05 indicate significant differences between the two specified groups.

In T6SS, immunity genes are generally tightly linked next to their isogenous effector genes, and the immunity proteins are produced to specifically bind and neutralize their cognate toxins [40]. Unexpectedly, downstream of *tle1*^*AH*^, there exists two open reading frames (ORF) with unknown function, AH17725 (*tli1*^*AH*^) and AH 17720 (*tli2*^*AH*^), both of which belong to the DUF2931 family. To determine which ORF plays an important role in protection against Tle1^AH^, we constructed three complementary plasmids that harbored the *tli1*^*AH*^ or *tli2*^*AH*^ gene alone or together and introduced these plasmids separately into the Δ*tle1*-*tli1tli2*^*AH*^ mutant (deletion of *tle1*^*AH*^ and its two cognate immunity genes). Using these mutants as preys and the wild-type NJ-35 and ∆*clpV* as predators, a competitive experiment was carried out. As shown in Figure 4, when ∆*clpV* was used as the predator strain, there exist no statistically significant differences in surviving prey cells between Δ*tle1*-*tli1tli2*^*AH*^ and its single restoration strain of *tli1*^*AH*^ or *tli2*^*AH*^, or between the single and double restoration strains, except for a significant difference between Δ*tle1*-*tli1tli2*^*AH*^ and the double restoration strain (*P *< 0.05). However, using the wild-type NJ-35 as the predator strain, the double restoration strain exhibits a significant surviving advantage over Δ*tle1*-*tli1tli2*^*AH*^ or the single restoration strains (*P* < 0.001), while no significant differences were observed between Δ*tle1*-*tli1tli2*^*AH*^ and its single restoration strains of *tli1*^*AH*^ or *tli2*^*AH*^. Compared with the ∆*clpV*, NJ-35 could cause substantial decreases in the number of surviving cells of Δ*tle1*-*tli1tli2*^*AH*^ and its single gene restoration strain of *tli1*^*AH*^ or *tli2*^*AH*^ (*P *< 0.01), but not make a significant difference in the surviving populations of the *tli1tli2*^*AH*^ double restoration strain. These data indicate that single gene restoration of *tli1*^*AH*^ or *tli2*^*AH*^ could not protect the ∆*tle1*-*tli1tli2*^*AH*^ mutant from antagonism by Tle1^AH^, and only when the two immunity proteins were coexpressed could the effective protection be conferred.

### Tle1^AH^ interacts with the VgrG protein

**Figure 5:**
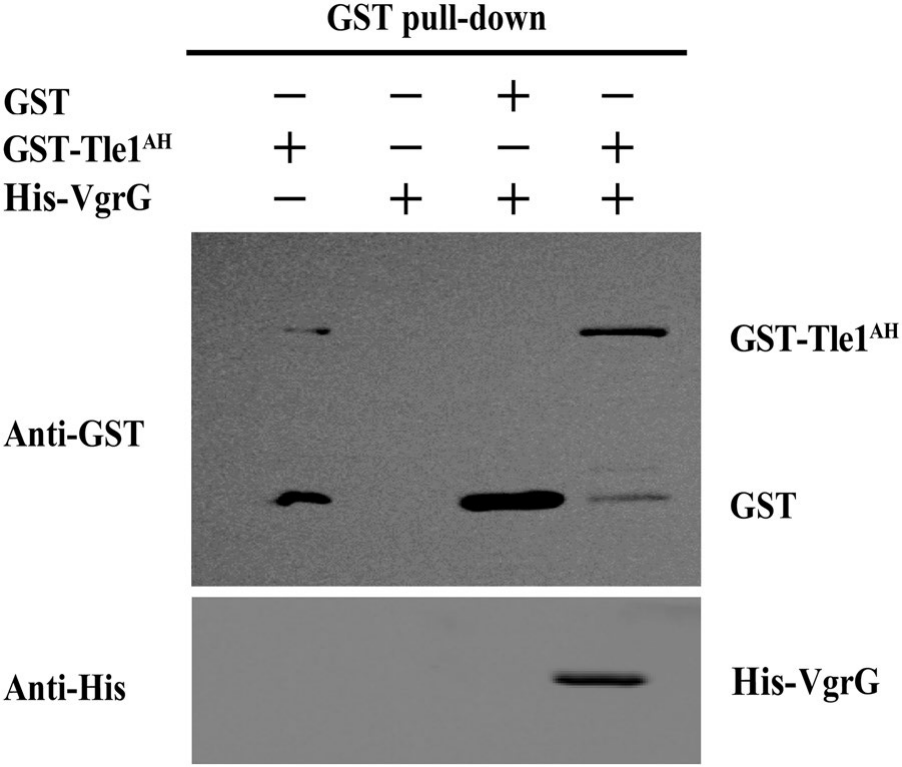
**The interaction of Tle1**^**AH**^**with VgrG was verified by an in vitro pull-down assay.** Purified GST, GST-Tle1^AH^ and His-VgrG were incubated with magnetic beads for the GST pull-down assay. Controls included the incubation with GST-Tle1^AH^ alone, His-VgrG alone, or GST with His-VgrG. Bound proteins were washed off the beads with the elution buffer and detected by immunoblotting using anti-GST or anti-His antibodies.

The VgrG and Hcp proteins have been proposed as carriers of T6SS effectors [19]. To gain insights into the secretion mechanism of Tle1^AH^, we investigated whether the VgrG or Hcp proteins encoded upstream of Tle1^AH^ were involved in Tle1^AH^ delivery. We tested the interactions of Tle1^AH^ with either VgrG or Hcp using a pull-down assay. Our results indicate that VgrG was able to bind to Tle1^AH^ (Figure 5), whereas Hcp failed to do so (data not shown), suggesting that VgrG is essential for Tle1^AH^ export into target cells.

### Tle1^AH^ is required for virulence and colonization of *A. hydrophila* NJ-35

**Figure 6:**
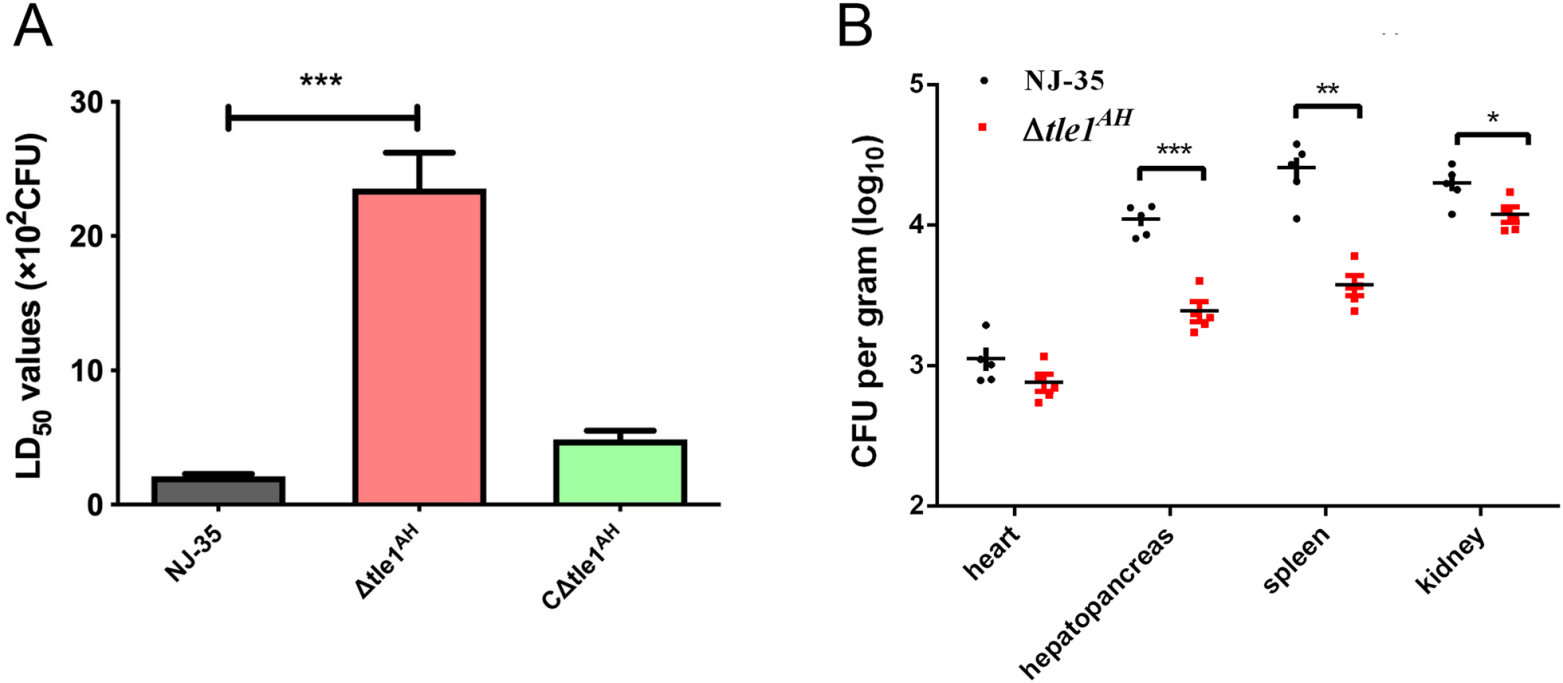
**Tle1**^**AH**^**is required for the virulence and colonization of*A. hydrophila*NJ-35. A** Determination of the LD_50_ values of the wild-type and *tle1*^*AH*^ mutant strains in zebrafish. Zebrafish were intraperitoneally (i.p.) injected with tenfold serially diluted bacterial suspensions. The control group was i.p. injected with sterile PBS only. **B** Competitive assays of NJ-35 and Δ*tle1*^*AH*^ in crucian carp. Strains were mixed at a ratio of 1:1 and inoculated to fish by intraperitoneal injection. After 24 h, heart, hepatopancreas, spleen and kidney were harvested for counting of the number of colony-forming units (CFU) per gram of sample. Data are presented as the mean ± standard deviation (error bars) of three independent experiments. ***P < 0.001, **P < 0.01, *P < 0.05.

Zebrafish is a well-established animal model to evaluate *Aeromonas* virulence [41]. To determine whether the *tle1*^*AH*^ gene affected bacterial virulence, the LD_50_ values of the wild-type and *tle1*^*AH*^ mutant strains were investigated using a zebrafish model. The LD_50_ value of *A. hydrophila* NJ-35 was 2.11 × 10^2^ CFU, while the *tle1*^*AH*^ mutant strain had an approximately 11-fold higher LD_50_ value than the wild-type strain (*P *< 0.001) (Figure 6A). Most dead fish showed typical clinical features of hemorrhagic septicemia. The results indicate that Tle1^AH^ affects the virulence of *A. hydrophila* NJ-35. Furthermore, we explored whether Tle1^AH^ was required for the colonization of the NJ-35 strain in crucian carp. As expected, the colonization ability of the ∆*tle1*^*AH*^ strain was significantly lower than that of the wild-type strain in the hepatopancreas (*P *< 0.001), spleen (*P *< 0.01) and kidney (*P *< 0.05) (Figure 6B). These data indicate that Tle1^AH^ facilitates the survival and colonization of *A. hydrophila* within the host, which is a common characteristic of antibacterial effectors.

### Tle1^AH^ is associated with biofilm formation of *A. hydrophila*

**Figure 7:**
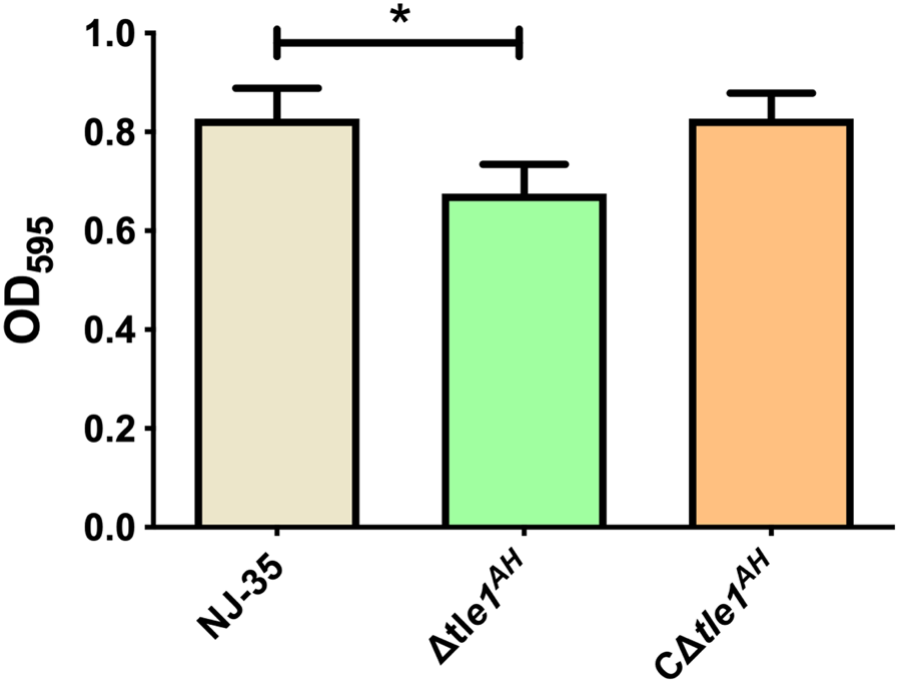
**Biofilm formation of the wild-type and*tle1***^***AH***^**mutant strains.** Biofilm formation was determined by crystal violet staining using 96-well plates, and the values were measured at OD_595_. Data are presented as the mean ± standard deviation (error bars) of three independent experiments. *P < 0.05.

To examine whether *tle1*^*AH*^ is involved in some biological characteristics of *A. hydrophila*, like the classical effector proteins Hcp or VgrG, we further assayed bacterial growth, motility and biofilm formation. Our results indicate that there were no significant changes in the growth rate (Additional file 2) and bacterial motility (Additional file 3) in the *tle1*^*AH*^ mutant compared to the wild-type strain. However, the biofilm formation phenotype of the ∆*tle1*^*AH*^ strain was weaker than that of the NJ-35 strain, and the decreased biofilm formation ability could be restored to the wild-type level in the C∆*tle1*^*AH*^ strain (Figure 7). This result suggests that in addition to antimicrobial activity, the *tle1*^*AH*^ gene may directly or indirectly influence the biofilm formation of *A. hydrophila.*

### Search for DUF4123 and downstream genes in *A. hydrophila* strains of the known T6SS components

**Figure 8:**
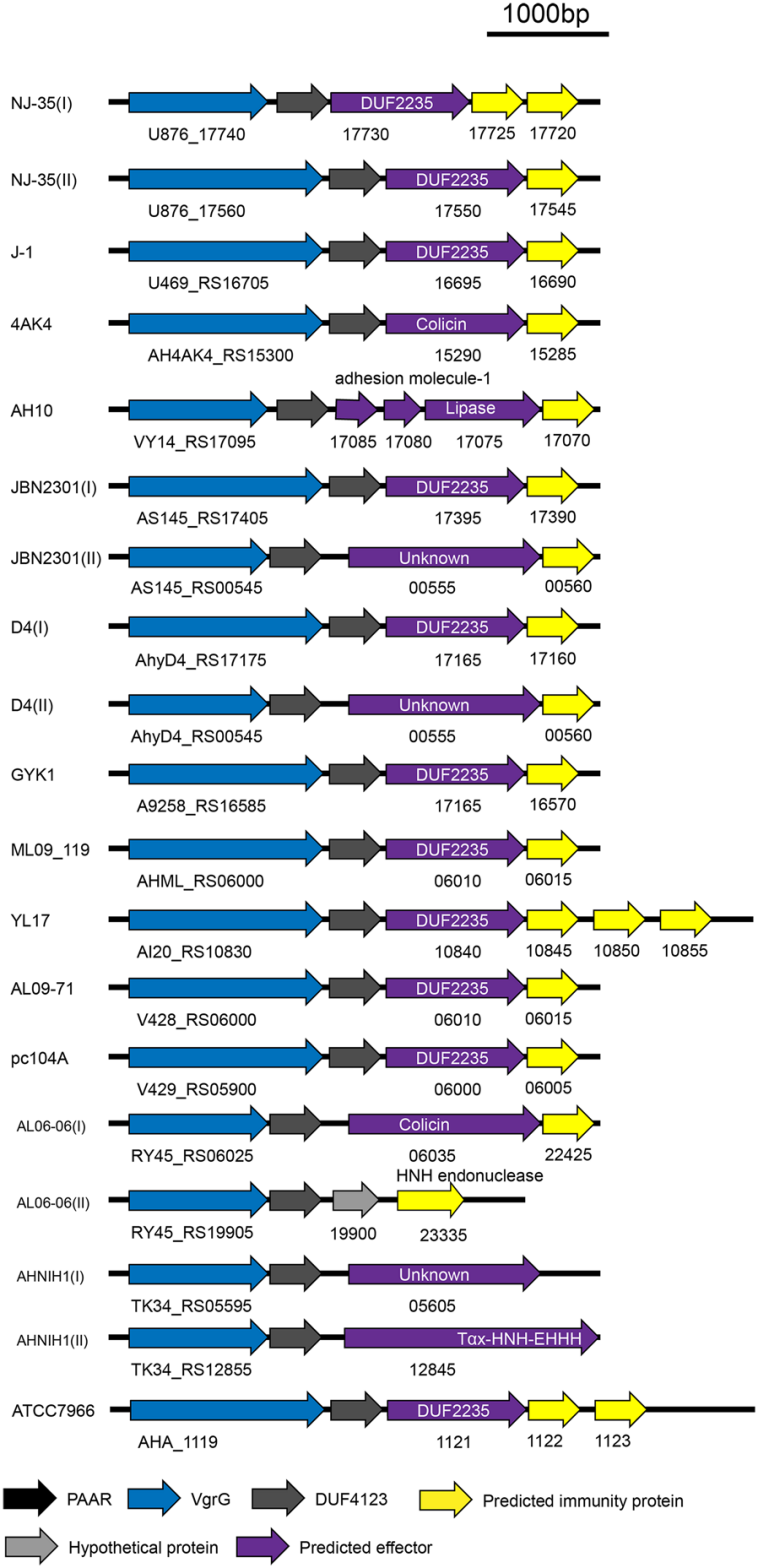
**Predicted DUF4123-associated T6SS effector-immunity (EI) gene modules in 14*A. hydrophila*strains.** All the strains used to search for putative effectors are listed in Table 2. The gray arrows indicate DUF4123, the purple arrows indicate the putative effectors, and yellow arrows indicate immunity genes. The arrows indicate the direction of transcription.

Given that the DUF4123 domain has been used to predict unknown T6SS effectors, we expanded our analyses for the large-scale search of effectors in *A. hydrophila* strains (Table 2). Many potential effectors were identified using DUF4123 as a signal (Figure 8). Most of the effectors encode the DUF2235 domain at the C-terminus and have a Gly-X-Ser-X-Gly motif, indicating that they are members of the Tle family. And genes encoding putative immunity proteins, which belong to the DUF2931 family, could be found downstream of their cognate effector genes. The number of immunity genes varies from none to three, indicating that E-I pairs exist in a variety of forms. In addition, there were also a few strains without the DUF2235 domain but with other colicin or unknown effector proteins.

**Table 2:** The *A. hydrophila* strains used to search for putative effectors

Strains	Accession number	Sequence range of DUF4123-immunity	Conserved domain of putative protein	Protein ID of putative protein
NJ-35	NZ_CP006870	3 862 649–3 866 785	DUF2235	WP_047234910
J-1	NZ_CP006883	3 759 517–3 763 012	DUF2235	WP_016349832
4AK4	NZ_CP006579	3 417 209–3 422 000	Colicin	WP_025327967
AH10	NZ_CP011100	3 826 164–3 830 669	Lipase	WP_045790457
JBN2301	NZ_CP013178	3 873 138–3 876 633	DUF2235	WP_016349832
		112 134–117 054	Unknown	WP_139118716
D4	NZ_CP013965	3 835 001–3 838 496	DUF2235	WP_016349832
		112 134–117 054	Unknown	WP_139118716
GYK1	NZ_CP016392	3 750 771–3 754 266	DUF2235	WP_016349832
ML09___119	NC_021290	1 315 162–1 318 657	DUF2235	WP_016349832
YL17	NZ_CP007518	2 331 025–2 336 071	DUF2235	WP_016349832
AL09_71	NZ_CP007566	1 314 833–1 318 328	DUF2235	WP_016349832
pc104A	NZ_CP007576	1 314 832–1 318 327	DUF2235	WP_016349832
AL06_06	CP010947	1 271 104–1 276 922	Colicin	WP_016349832
		4 374 167–4 376 145	HNH endonuclease	WP_016349832
AHNIH1	NZ_CP016380	1 185 505–1 190 948	Unknown	WP_016349832
		2 892 361–2 897 678	Tox-HNH-EHHH	WP_016349832
ATCC 7966^T^	NC_008570	1 215 532–1 219 858	DUF2235	WP_016349832

## Discussion

To adapt to the complexity of the living environment, microbes have evolved many mechanisms to compete with other species for limited nutrition [42–44]. Some Gram-negative bacteria encode T6SS weapons that deliver toxins to either prokaryotic or eukaryotic cells to mediate different signals [45]. Many studies have successfully identified effector proteins using diverse methods [21, 24, 46]. In this study, we predicted a T6SS effector protein, AH17730, in the genome of *A. hydrophila* NJ-35 based on the conserved domain DUF4123. By analyzing the amino acid sequence of AH17730, we identified a catalytic motif Gly-X-Ser-X-Gly, which can be found in the Tle lipase family. Tle proteins have been reported as a superfamily of T6SS phospholipase effectors that directly target the cell membrane by hydrolyzing its lipid components [47]. The known Tle proteins are classified into five groups according to their protein sequences and phylogenetic distribution. In this study, we demonstrate that AH17730 has phospholipase activity and belongs to the Tle1 family; therefore, AH17730 was named Tle1^AH^.

Previously, Tle1^BT^ was shown to be required for the antibacterial activity of *Burkholderia thailandensis* [47]. Similarly, our study demonstrates that Tle1^AH^ is essential for *A. hydrophila* to kill *E. coli* or *V. parahaemolyticus* in a T6SS-dependent manner. Notably, the antibacterial competition experiment shows that Tle1^AH^ could not act on closely related strains or species, such as *A. hydrophila* strains ATCC 7966, J-1 and NJ-3, *A. salmonicida* CS-2, *A. media* NJ-8 and *A. veronii* XH-14. For this reason, we speculated that it might be related to cognate immunity proteins. In our work, we identified two putative immunity proteins, AH17725 and AH17720, downstream of Tle1^AH^, and bioinformatics analysis shows that the two proteins belong to the DUF2931 superfamily. Some members of this superfamily have been annotated as outer membrane lipoproteins, which is consistent with the localization of Tle1^AH^. Functional analysis indicates that the two immunity proteins were required to work together to protect *A. hydrophila* NJ-35 against toxicity of Tle1^AH^. To further determine whether a correlation exists between the toxicity of Tle1^AH^ and the distribution of immune genes, we searched for the known genomes of two *A. hydrophila* strains, including ATCC 7966 (accession number NC_008570), a well-characterized type strain for the species, originally isolated from “a tin of milk with a fishy odor” [48], and J-1 (accession number CP006883), an epidemic piscine strain of China [49]. As expected, the genome of ATCC 7966 contains the same E-I module (AHA_1121-1123) as AH17720-17730 of NJ-35. In the J-1 genome, a gene that has 100% identity with *tle1*^*AH*^, V469_RS16695, was found, and a DUF2931 family protein is encoded downstream of this gene. Therefore, it is possible that the same or similar immune proteins in the above *Aeromonas* strains play a role in resisting the toxicity of Tle1^AH^ from NJ-35. Unfortunately, no complete genome sequences are available for strains NJ-3, CS-2, NJ-8 or XH-14. A recent study indicated that an additional non-native T6SS auxiliary cluster can be acquired and used by a *V. cholerae* strain to kill kin cells lacking the immunity protein [50]. Whether such evolutionary dynamics of the *V. cholerae* T6SS actually exist in *Aeromonas* will be the subject of our future study.

Interestingly, our results show that only when two immunity proteins worked together could *A. hydrophila* NJ-35 assure self-protection against toxicity of Tle1^AH^. Similar findings have not been previously reported. Although it is not surprising that two homologous immunity genes could be found to coexist downstream of the *tle1* gene, expression of the immunity gene that is most closely adjacent to *tle1* is usually sufficient to abolish Tle1 activity. A previous study on the entero-aggregative *E. coli* (EAEC) Sci-1 T6SS indicated that the *tle1*^*EAEC*^ gene, which encodes an effector and is responsible for Sci-1-mediated antibacterial activity, is followed by a duplicated region encoding two putative immunity proteins, EC042_4535 and EC042_4536; the production of EC042_4535 (Tli1^EAEC^) in *∆EC042_4535*-*4536* prey cells protected themselves against EAEC killing, and the Tle1^EAEC^ activity was completely abolished with a Tle1^EAEC^:Tli1^EAEC^ molecular ratio of 1:1 [51]. We do not know much about the binding ratios of the Tle1 effector and its cognate immunity proteins in various bacteria. It is possible that future structure-based functional analysis of Tle1 and its complexes with immunity proteins will reveal the diverse mechanisms of inhibition by immunity proteins.

Different mechanisms have been proposed or identified for cargo effectors. They can directly or indirectly contact the VgrG spike, the Hcp rings or PAAR proteins [16, 52]. Genes coding for Tle superfamily proteins, which are known as cargo effectors, are commonly found in the vicinity of the *vgrG* gene [18, 47]. Therefore, one may hypothesize that these effectors will be transported by interaction with VgrG. To determine the Tle1^AH^ secretion mechanism, in this work, we performed a pull-down assay and demonstrated that Tle1^AH^ could interact with VgrG but not Hcp. We speculate that this interaction was required for proper Tle1^AH^ delivery. In a previous study, the transport of Tle1^EAEC^ was thought to be required for binding the C-terminal extension of VgrG1 in *E. coli*, and a putative protein–protein interaction module of this extension revealed a transthyretin-like (TTR) domain fold [51]. For the T6SS-mediated delivery of a given VgrG-binding Tle1, multiple binding events likely occur in a certain order that includes Tle1 binding to the cognate VgrG and to the immunity protein. In the process, we do not know whether there are other chaperone proteins involved and how the DUF4123 domain functions. In the future, structural analyses of the DUF4123, VgrG, and Tle1 proteins are required to fully understand the mechanisms of Tle1 delivery.

Although the T6SS plays a significant role in antimicrobial competition, there remains a possibility that it serves some purposes beyond competition. PA2374, an effector secreted by H3-T6SS of *Pseudomonas*, is important for iron uptake and functions by interacting with outer membrane vesicles and the *Pseudomonas* quinolone signal system [53]. It has also been reported that the VgrG and Hcp proteins are also involved in motility, protease activity, biofilm formation and virulence in addition to competitive growth in *A. hydrophila* SSU (now belonging to *Aeromonas dhakensis* species) [54]. In the present study, we demonstrate that the *tle1*^*AH*^ gene was involved in biofilm formation and virulence, and influenced the colonization ability of *A. hydrophila* NJ-35 in crucian carp. The findings indicate that the effects of Tle1^AH^ on *A. hydrophila* virulence are multidimensional, which also explains the complexity of the pathogenic mechanism of this bacterium.

In this study, we identified a Tle1^AH^ effector protein of T6SS in *A. hydrophila* NJ-35, with two cognate immunity proteins working together to prevent sibling bacteria from intoxication. Also, we identified some putative Tle1 family effectors in *A. hydrophila* strains with known genome sequences, and interestingly, these strains have been determined to be virulent [55–59], implying that Tle1 may be related to *A. hydrophila* virulence. Hopefully, there are more diverse effectors to be identified, which will provide a deeper understanding of the T6SS strategies of *A. hydrophila.*

## Supplementary information

**Additional file 1. Primers used in this study.** Underlined sequences indicate restriction sites.

**Additional file 2. Growth curve of the wild-type and*tle1***^***AH***^**mutant strains.** The cells were cultured in LB broth and densities were measured every 2 h at OD_600_. Data are presented as the mean ± standard deviation (error bars) of three independent experiments.

**Additional file 3. Motility of the wild-type and*tle1***^***AH***^**mutant strains.** Swimming ability was observed after culturing strains at 28 °C for 48 h on 0.3% LB agar plates. The migration diameters were measured to assess the motility. Data are presented as the mean ± standard deviation (error bars) of three independent experiments.

## Data Availability

All data generated or analysed during this study are included in this published article and its additonal files.

## Abbreviations

DUF: domain of unknown function
Tle1: type VI lipase effectors
VgrG: valine-glycine repeat protein G
Hcp: hemolysin co-regulated protein
PAAR: proline-alanine-alanine-arginine
IPTG: isopropyl β-d-1-thiogalactopyranoside
LB: Luria–Bertani
OD: optical density
PBS: phosphate buffered saline
Cm: chloramphenicol
Amp: ampicillin
Kan: kanamycin
Gm: gentamicin
PCR: polymerase chain reaction
CFU: colony forming unit
SDS-PAGE: sodium dodecyl sulfate–polyacrylamide gel electrophoresis
TCA: trichloroacetic acid
GroEL: heat shock protein Hsp60
LD_50_: median lethal dose
